# Antibiotic-Induced Pulmonary Fibrosis: National Database Analysis

**DOI:** 10.3390/biomedicines14061182

**Published:** 2026-05-22

**Authors:** Olga Butranova, Yury Kustov, Anna Abramova, Sergey Zyryanov, Irina Asetskaya, Elizaveta Terekhina, Vitaly Polivanov

**Affiliations:** 1 Department of General and Clinical Pharmacology, Peoples’ Friendship University of Russia Named After Patrice Lumumba (RUDN), 6 Miklukho-Maklaya St., 117198 Moscow, Russia; butranova-oi@rudn.ru (O.B.); abramova-annand@rudn.ru (A.A.); zyryanov-sk@rudn.ru (S.Z.); asetskaya-il@rudn.ru (I.A.); terekhina-enk@rudn.ru (E.T.); 2 Moscow City Health Department, City Clinical Hospital No. 24, State Budgetary Institution of Healthcare of the City of Moscow, Pistzovaya St. 10, 127015 Moscow, Russia; 3 Pharmacovigilance Center, Information and Methodological Center for Expert Evaluation, Record and Analysis of Circulation of Medical Products Under the Federal Service for Surveillance in Healthcare, 4-1 Slavyanskaya Square, 109074 Moscow, Russia; pvit74@gmail.com

**Keywords:** pulmonary fibrosis, antibiotics, trimethoprim-sulfamethoxazole, azithromycin, levofloxacin, doxycycline, cefuroxime

## Abstract

**Background**: Pulmonary fibrosis (PF) is a major global health issue associated with substantial morbidity across all age groups. One of the important etiological factors contributing to PF is drug-induced lung injury, which can result from both direct and indirect damage to the pulmonary parenchyma caused by various pharmacological agents, including chemotherapeutics, antirheumatic drugs, cardiovascular medications, and certain antimicrobial agents. The aim of our study was to assess the structure of antibacterials involved in drug-induced PF (DIPF) and analyze signals of DIPF, calculating the reporting odds ratio (ROR) and proportional reporting ratio (PRR) using spontaneous reports (SRs) extracted from the Russian National Pharmacovigilance database. **Methods**: A retrospective, descriptive pharmacoepidemiological analysis of SRs from the AIS database for the period 1 April 2019–31 March 2025 was conducted. **Results**: A total of 130 SRs with data on DIPF associated with antibacterial agents were identified, with patients’ mean age of 59.1 ± 14.46 years. Death was reported in 65 SRs (50%) with a mean age of 53.0 ± 13.66 years. Next, antibacterials were identified as leaders: sulfamethoxazole (used alone or in combination with trimethoprim, 20.7% (n = 50)), azithromycin (18.2%, n = 44), levofloxacin (12.4%, n = 30), doxycycline (11.6%, n = 28), and cefuroxime (10.7%, n = 26). Disproportionality analysis performed with PRR and ROR calculation revealed the strongest association with DIPF for cefuroxime (PRR = 15.11, 95% confidence interval, CI: 10.25–22.27; ROR = 15.31, 95% confidence interval, CI: 10.33–22.68). **Conclusions**: Cefuroxime was revealed as a drug with an unexpected but robust safety signal for DIPF, warranting heightened clinical awareness and further investigation. The observed associations between antibacterial agents and DIPF should be interpreted with caution, as they may reflect protopathic bias (antibiotics prescribed for early symptoms of unrecognized pulmonary fibrosis) or context-dependent biological effects rather than true pro-fibrotic drug properties. Our findings do not establish causality but rather generate safety signals that warrant validation through prospective studies with detailed clinical phenotyping and mechanistic investigations using human cell lines.

## 1. Introduction

A substantial proportion of non-infectious respiratory disorders that impose significant disability can be attributed to fibrosing interstitial lung disease (ILD). Idiopathic pulmonary fibrosis (PF) is the most common form of ILD with an unknown precise etiology; other forms include different variants of interstitial pneumonia and pneumonitis, which may be induced by hypersensitivity reactions [1], may have an autoimmune origin [2], or may be a result of exposure to various exogenous factors, including drugs [3].

A 23-year analysis of the Food and Drug Administration Adverse Event Reporting System (FAERS) database found that drug-induced pulmonary fibrosis (DIPF) reports constitute approximately 0.07% of all reported adverse events (AEs) [3]. An analysis of the Russian national pharmacovigilance database demonstrated that the annual number of spontaneous reports (SRs) documenting DIPF nearly doubled from 2021 to 2022, and again from 2022 to 2023, reaching a total of 477 cases in 2023 [4].

According to the data derived from the FAERS database, the top-five drug classes associated with DIPF were antirheumatic drugs (39.4%), antineoplastic agents (26.4%), cardiovascular drugs (12.6%), corticosteroids (4.6%), and immunosuppressants (4.0%) [3]. Russian data indicated the same pharmacological classes: antineoplastic and immunomodulatory agents were the main culprit groups [4]. In the study performed by Jiang T et al. (2024), antineoplastic agents were shown to be the most common cause of lung damage in all age groups, regardless of gender, while cardiovascular agents and antirheumatic drugs ranked second and third, respectively, and the fourth most common group was antibacterial agents [5]. The specified drug classes possess mechanisms of action that may contribute to their potential to induce damage to the lung parenchyma. Direct cytotoxicity is characteristic not only of antineoplastic agents but also of antibacterials. The last group was shown to be involved in the development of various patterns of pulmonary toxicity (e.g., organizing pneumonia for minocycline; PF for nitrofurantoin; and eosinophilic pneumonia for minocycline and nitrofurantoin) [6]. Published literature identifies amphotericin B, isoniazid, nitrofurantoin, sulfasalazine, and other antibacterials as agents whose use has been associated with interstitial lung injury and subsequent development of PF [7,8,9].

Antibiotic-induced PF emerges through two interrelated pathophysiological pathways: direct cytotoxicity and immune-mediated injury, both ultimately converging on fibroblast activation and excessive extracellular matrix (ECM) deposition [10]. In the cytotoxic pathway, drugs such as nitrofurantoin and bleomycin generate reactive oxygen species (ROS)—including superoxide anion (O_2_^−^), hydrogen peroxide (H_2_O_2_), and hydroxyl radical (-OH)—through redox cycling in oxygen-rich pulmonary tissue. Nitrofurantoin undergoes nitroreduction by cellular reductases, producing toxic metabolites that cause lipid peroxidation, protein denaturation, and DNA damage in alveolar epithelial cells, while simultaneously depleting antioxidant defenses (glutathione and catalase) and inducing lymphocytic alveolitis [11]. Bleomycin, by forming an iron–drug complex (bleomycin–Fe^2+^), directly cleaves DNA in type II pneumocytes; at the cellular level, this elevates both mitochondrial and cytoplasmic ROS, upregulates a pro-fibrotic gene network, and induces mitochondrial swelling and dysfunction in lung fibroblasts [12]. Oxidative stress initiates the core fibrogenic signaling cascade, wherein ROS-mediated activation of transforming growth factor-β1 (TGF-β1) leads to phosphorylation of critical signaling components, SMAD family member 2 and SMAD family member 3 (SMAD2 and SMAD3). Phosphorylated SMADs drive transcriptional upregulation of collagen type I alpha 1 (COL1A1), alpha smooth muscle actin (α-SMA), and fibronectin 1 (FN1). This process mediates myofibroblast differentiation and the irreversible accumulation of ECM [13]. Macrophage polarization towards the M2 phenotype represents an additional amplifying mechanism—bleomycin-conditioned macrophages secrete TGF-β1, interleukin-6 (IL-6), and fibronectin, which are sufficient to induce systemic fibrosis independently of direct drug exposure [14]. In parallel, an immune-mediated pathway accounts for idiosyncratic responses: drug metabolites (e.g., those of moxifloxacin) act as haptens, forming antigenic complexes that activate T-cell-dominated delayed-type hypersensitivity (type IV Gell–Coombs) [15]. Cytokine release—particularly TGF-β1 and tumor necrosis factor-alpha (TNF-α)—tips the microenvironment towards a pro-fibrotic state, while nitrofurantoin additionally induces a proto-myofibroblastic phenotype in lung fibroblasts characterized by enlarged cell area, decreased roundness, and increased fibronectin deposition at concentrations of ≥20 μg/mL [16]. The chronicity of fibrosis is further perpetuated by a self-reinforcing loop: sustained ROS production potentiates TGF-β1 expression, which in turn stimulates further ROS generation, driving progressive and often irreversible remodeling of the pulmonary parenchyma [11].

Antineoplastic agents are primarily utilized by patients with oncological conditions; in contrast, antibiotics represent some of the most widely used medications across the general population [17,18,19]. This disparity highlights the critical importance of rigorously investigating all safety-related factors, including rare AEs such as DIPF, which may accompany use of antibiotics. FAERS database analysis performed by Jiang T et al. (2024) revealed a significant contribution of antibacterial agents in DIPF development in the following age groups: 0–6 years (13.95%), 13–18 years (13.25%), and 19–40 years (10.69%) [5]. This points to the relevance of studying the issue of antibiotic-induced PF. The aim of our study was to estimate the structure of antibacterials involved in DIPF and analyze signals of DIPF, calculating ROR and PRR using SRs extracted from the Russian National Pharmacovigilance database.

## 2. Materials and Methods

**Figure 1:**
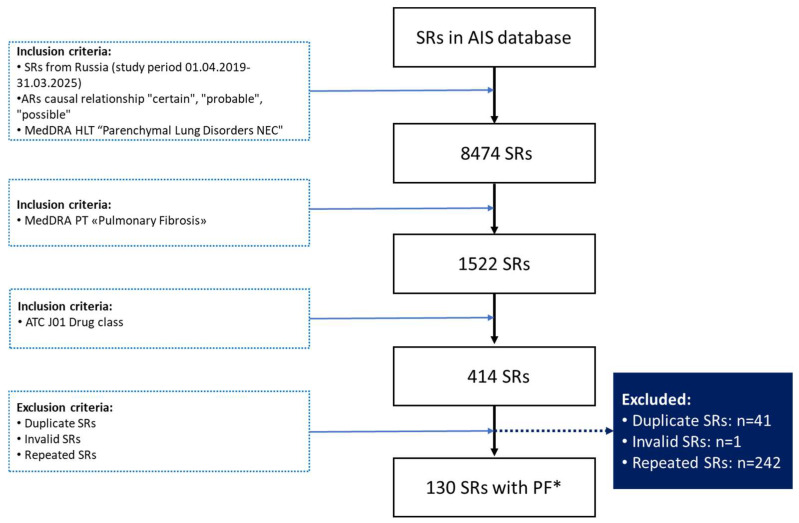
Flowchart of SRs selection from the AIS “Pharmacovigilance” (AR—adverse reaction; HLT—high-level term; PT—preferred term; ATC—anatomical therapeutic chemical classification System; SR—spontaneous report). *—including information from 242 repeated SRs. Note: Duplicate SRs denote two or more unsolicited communications that describe the same suspected AR(s) in the same patient, associated with the same medicinal product(s), but originating from distinct, independent sources (for example, a healthcare professional and a patient, two different healthcare professionals, or a regulatory authority and a marketing authorisation holder). Repeated SRs denote instances in which the same source (for example, the original reporter, a published article, or an electronic transmission) submits identical or substantially similar information about a single case on multiple occasions; this may occur through inadvertent re-transmission of an initial or follow-up report, submission of a follow-up that contains no new clinically significant information (a null follow-up), or repeated unsolicited contacts from the same reporter providing substantially similar or additional facts.

Data on AEs in the Russian Federation is collected by the central pharmacovigilance body, the Federal Service for Surveillance in Healthcare (Roszdravnadzor), utilizing the AIS “Pharmacovigilance” database, which is designed and maintained in accordance with the ICH E2B (R3) standard [20]. MedDRA version 28.1 is used in the AIS [21] and the built-in WHO algorithm, as well as the Naranjo algorithm [22]. Reporting can be carried out by healthcare professionals, pharmaceutical industry staff, patients, and their representatives. The data source for our study was the AIS database. The study design is a retrospective, descriptive pharmacoepidemiological analysis of SRs recorded in the AIS database for the period 1 April 2019–31 March 2025. Adverse drug reactions (ADRs) underwent causality assessment utilizing the Naranjo algorithm, and only those categorized as “certain,” “probable,” or “possible” were incorporated into the final dataset for analysis. Drug identification in SRs was made using the Anatomic Therapeutic Chemical Classification system (ATC). For inclusion, SRs originating from Russia or other countries had to contain the MedDRA Preferred Term (PT) “Pulmonary fibrosis” and at least one antibacterial agent for systemic use classified under ATC code J01. From this initial cohort, duplicate and invalid reports were removed. The validation process was conducted in accordance with paragraph 407 of the “Rules of Good Pharmacovigilance Practice of the EAEU” [23], which mandates the presence of four core elements: an identifiable reporter, an identifiable patient, at least one suspected medicinal product, and at least one suspected ADR. Any SR lacking one or more of these elements was deemed invalid. The authors further evaluated the completeness of information pertaining to the suspected drug, the ADR, patient, and reporter data. The data-collection process included the identification and estimation of repeated SRs. Repeated SRs were defined as reports submitted by the same reporting source that contained identical or near-identical information regarding the same case on more than one occasion. When repeated SRs contained new, clinically relevant information, those details were incorporated into the analysis. Where possible, data items were cross-checked across SRs to resolve inconsistencies and to distinguish independent duplicate SRs from true repeated SRs originating from the same source. Duplicate SRs were defined as SRs made by two or more independent reporters describing the same suspected AR(s) in the same patient, associated with the same medicinal product(s). The severity of the ADR was classified according to paragraph 2 of the EAEU guidelines [23]. Figure 1 discloses the process of SRs selection from the AIS database.

Within the framework of our study, suspected medications were first identified by their International Nonproprietary Names (INN) and then categorized using ATC groups. Demographic information was derived from the SRs, and all data processing was conducted in Microsoft Excel 2019. The statistical approach was descriptive; qualitative parameters were summarized by their absolute and relative frequencies (n, %), while the mean, standard deviation, median, interquartile range (IQR), and the first and third quartiles (Q1, Q3) were computed for all quantitative parameters. It is important to note that statistical significance testing was not performed, as the methodology of analyzing spontaneous reports does not support making inferences about the total population of medication users.

To identify potential safety signals, a disproportionality analysis was performed using ROR and PRR as the main frequency methods. In the field of pharmacovigilance, ROR serves as a quantitative measure of the association strength between a drug and AE. It is derived by comparing the frequency of reporting for a specific drug-AE pair against the frequency observed for other drug-AE combinations. ROR value exceeding 1 suggests a heightened rate of reporting for the given association. PRR compares the proportion of a specific AE linked to a particular drug with the proportion of the same AE reported for all other drugs within a database. A PRR value greater than 1, accompanied by a lower bound of the 95% confidence interval (CI) also exceeding 1, indicates disproportionate reporting of the AE for the drug in question, thereby signaling a potential safety issue warranting further evaluation. The minimum criteria for a signal detection (AE considered as ADR) recommended for PRR are as follows: PRR ≥ 2, number of cases ≥ 3, chi-squared (χ^2^) ≥ 4 [24,25]. Minimum criteria for ROR: ROR > 1, lower bound of 95% CI > 1 [24,25].

For disproportionality analysis, we used a 2 × 2 contingency table (Table 1).

The PRR was calculated using the following formula:

$$PRR=\frac{\frac{a}{a+b}}{\frac{c}{c+d}}$$

The 95% confidence interval (CI) for PRR was also calculated:

$$95\%{CI}_{PRR}=e^{\ln\left(PRR\right)\pm 1.96*\sqrt{\frac{1-\frac{a}{a+b}}{a}+\frac{1-\frac{c}{c+d}}{c}}}$$

We considered a signal detected if the following criteria were met: PRR ≥ 2, a chi-squared (χ^2^) statistic ≥ 4, a minimum of three cases (a ≥ 3), and a lower bound of the 95% CI greater than 1.

The ROR was calculated as the odds of a specific reaction occurring with the suspected drug compared to its odds with all other drugs:

$$ROR=\frac{a*d}{b*c}$$

The 95% confidence interval (CI) for the ROR was calculated using Woolf’s method:

$$95\%CI=e^{\ln\left(ROR\right)\pm 1.96*\sqrt{\frac{1}{a}+\frac{1}{b}+\frac{1}{c}+\frac{1}{d}}}$$

A disproportionality safety signal (association between a specific ATC J01 drug and DIPF) was considered detected if the lower bound of the 95% CI for ROR exceeded 1, and if there were at least three individual case reports for a drug–event combination of interest (a ≥ 3) [26].

We used the following definitions in our study [27].

“Adverse reaction—A response to a medicinal product, which is noxious and un-intended. Adverse reaction may arise from use of the product within or outside the terms of the marketing authorization or from occupational exposure. Use outside the marketing authorization includes off-label use, overdose, misuse, abuse, and medication errors”.

“Causality—In accordance with ICH-E2A, the definition of an adverse reaction im-plies at least a reasonable possibility of a causal relationship between a suspected medicinal product and an adverse event. An adverse reaction, in contrast to an adverse event, is characterized by the fact that a causal relationship between a medicinal product and an occurrence is suspected. For regulatory reporting purposes, as detailed in ICH-E2D, if an event is spontaneously reported, even if the relationship is unknown or unstated, it meets the definition of an adverse reaction. Therefore, all spontaneous reports notified by healthcare professionals or consumers are considered suspected adverse re-actions, since they convey the suspicions of the primary sources, unless the reporters specifically state that they believe the events to be unrelated or that a causal relationship can be excluded”.

“A SR is an unsolicited communication by a healthcare professional, or consumer to a competent authority, marketing authorisation holder or other organization (e.g., regional pharmacovigilance center, poison control center) that describes one or more suspected adverse reactions in a patient who was given one or more medicinal products. It does not derive from a study or any organized data collection systems”.

## 3. Results

**Figure 2:**
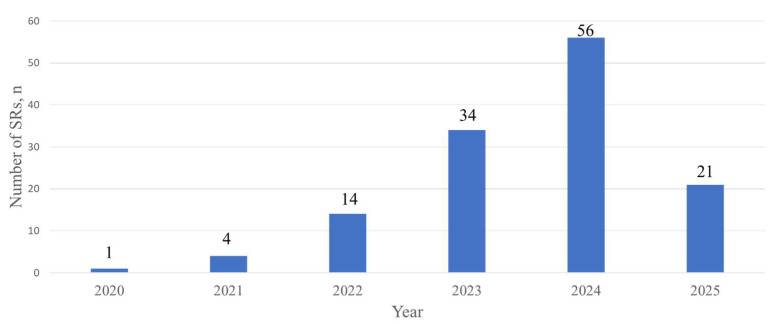
Annual SR distribution.

The total number of SRs referred to MedDra high-level term “Parenchymal lung disorder NEC” was 8474. The final number of SRs with PF related to antibacterials for systemic use after duplicates and invalid SRs exclusion was 130 (10.2%). The distribution of the number of SRs per year of reporting is presented in Figure 2. The dynamics revealed a steady trend of an increase in the annual number of SRs.

Outcome analysis based on the SRs data indicated a high prevalence of lethal cases (n = 65; 50%). Detailed information is given in Table 2.

In the sample of SRs with lethal outcomes, the following demographic features were reported. Males were 20% (n = 13), females were 78.5% (n = 51), and those with no data on gender were 1.5% (n = 1). Mean age was 53.0 ± 13.66 (min = 43, max = 97) years, the median was 44 (Q1 = 43; Q3 = 65; IQR = 22) years, and no data on age was xin 7.7% (n = 5) SRs.

The same predominance of females over males (63.1% (n = 82) vs. 35.4% (n = 46)) was revealed in the total sample of SRs. No data on gender was in 1.5% (n = 2). The mean age was 59.1 ± 14.46 (min = 31 years, max = 97) years, the median was 65 (Q1 = 44; Q3 = 66; IQR = 22) years, and no data on age was found in 3.8% (n = 5). In the total sample, 44.6% (n = 58) were adults, and 55.4% (n = 67) were elderly patients. No pediatric SRs were detected.

Time to onset was recorded in only 17.7% of SRs, dechallenge information in 13.8%, and rechallenge in 0.

### 3.1. The Structure of Antibacterial Drugs Involved in PF

The mean number of antibacterials of the group J01 per SR was 2.2 ± 1.5 (min = 1, max = 9; median = 1; Q1 = 1, Q3 = 3, IQR = 2). Notably, the majority of SRs (50.8%) involved only a single antibacterial drug, establishing a clear causal relationship. Detailed data is shown in Table 3.

The total number of suspected J01 drugs was 242. There were 8 ATC J01 subgroups detected in our study, with sulfonamides and trimethoprim ranking first in prevalence. The structure of J01 subgroups is given in Table 4. The detailed list of drugs revealed within each subgroup is demonstrated in Table S1. The main characteristics of the top-five subgroups of J01 drugs involved in DIPF will be discussed below.

**Figure 3:**
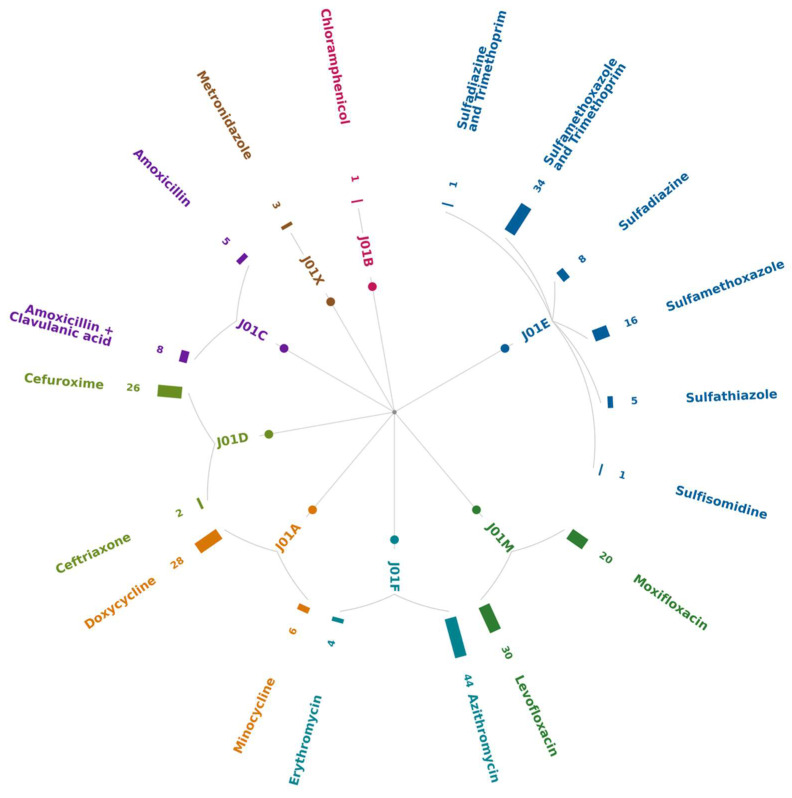
Antibacterial agents identified in SRs with DIPF.

Our study revealed a total of 18 different antibacterial agents (Figure 3). The most prevalent was sulfamethoxazole (used alone or in combination with trimethoprim), which accounted for up to 20.7% (n = 50) in the total structure of revealed antibacterials. Other antibacterials with the highest rates of occurrence were azithromycin (18.2%, n = 44), levofloxacin (12.4%, n = 30), doxycycline (11.6%, n = 28), and cefuroxime (10.7%, n = 26). Detailed data on involved drugs are presented in Table S1 and Figure 3. It is important to note that the frequency of occurrence in SRs reflects reporting patterns and should not be equated with signal strength. Disproportionality analysis (PRR, ROR) was performed to identify statistical signals of disproportionate reporting; the results are given in the corresponding section.

### 3.2. J01E Group

Sulfonamides and trimethoprim were the most prevalent J01 subgroup identified in our study. In the sample of SRs containing data on DIPF and drugs of the J01E group, female gender was reported in 70.8% (n = 46), male in 23.1% (n = 15), and no data was in 6.2% (n = 4). Mean age was 55.6 ± 15.5 (min = 43, max = 97, median = 48.5, Q1 = 43, Q3 = 65, and IQR = 22) years. Outcome assessment revealed a significant proportion of such outcomes as death (63.1%, n = 41), an unknown outcome was reported in 27.7% (n = 18) of SRs, condition unchanged in 7.7% (n = 5), and recovery without consequences in 1.5% (n = 1).

Six different drugs were revealed within the J01E group; information is given in Table 5.

The combination of sulfamethoxazole with trimethoprim, as well as sulfamethoxazole used alone, were the most prevalent agents within group J01E, constituting up to 76.9% (n = 50). The following demographic characteristics were derived from SRs containing data on these drugs: females were 82% (n = 41), males were 14% (n = 7), and no data on gender was reported in 4% (n = 2). Mean age was 52.2 ± 15.2 (min = 43, max = 97, median = 65, Q1 = 43, Q3 = 65, and IQR = 22) years. Lethal outcome was the most common (68%, n = 34), followed by unknown (22%, n = 11), and condition unchanged (10%, n = 5).

### 3.3. J01M Group

Quinolone antibacterials ranked second in the overall structure (20.7%, n = 50). Analyzing SRs, we revealed males in 86% (n = 43), females in 12% (n = 6), and absence of age data in 2% (n = 1). Mean age was 64.9 ± 7.9 (min = 44, max = 80, median = 65, Q1 = 65, Q3 = 65, IQR = 0) years. Unknown outcomes were the most common (64%, n = 32), followed by death (36%, n = 18).

There were only two drugs in this subgroup: levofloxacin (60%, n = 30) and moxifloxacin (40%, n = 20). Based on data in SRs containing levofloxacin, we identified an absolute predominance of males (97%, n = 29); females were only 3% (n = 1). The mean age was 67.0 ± 4.2 (min = 65, max = 80, median = 65, Q1 = 65, Q3 = 65, and IQR = 0) years. Unknown outcome predominated (73.3%, n = 22), and in 26.7% (n = 8), SR death was reported. Analysis of SRs containing data on moxifloxacin revealed that females were 30% (n = 6), males were 70% (n = 14), the mean age was 61.8 ± 10.7 (min = 44, max = 75, median = 65, Q1 = 50, Q3 = 66.75, and IQR = 16.75) years. Half of the outcomes were lethal (n = 10), and half were unknown (n = 10).

### 3.4. J01F Group

Macrolides, lincosamides, and streptogramines were the third most common subgroup revealed in our study (19.8%, n = 48). Male gender was reported in 56.3% (n = 27) of SRs, female was in 41.7% (n = 20), and no data on gender was in 2.1% (n = 1). Mean age was 68 ± 11 (min = 31 years, max = 80 years, median = 65, Q1 = 65, Q3 = 78, IQR = 13) years. Most SRs were marked with unknown outcome (60.4%, n = 29), death was detected in 22.9% (n = 11), condition unchanged in 14.6% (n = 7), and recovery without consequences in 2.1 (n = 1). There were only two drugs in the J01F group: azithromycin (91.7%, n = 44) and erythromycin 8.3% (n = 4). Among SRs containing azithromycin, males were 61.4% (n = 27), females were 36.4% (n = 16), and no data on gender was found in 2.2% (n = 1). Mean age was 70.2 ± 8.6 (min = 31, max = 80, median = 69, Q1 = 65, Q3 = 78, IQR = 13) years.

### 3.5. J01A Group

Tetracyclines ranked fourth in the structure of ATC J01 agents revealed in our study (n = 34, 14%). According to the data derived from SRs, males were 97.1 (n = 33), females were only 2.9% (n = 1). Mean age was 45.2 ± 6.1 (min = 43, max = 80, median = 43, Q1 = 43, Q2 = 44, IQR = 1) years. In the structure of outcomes, death was the leader (67.6%, n = 23), followed by condition unchanged (20.6%, n = 7), recovery without consequences (5.9%, n = 2), and unknown outcome (5.9%, n = 2).

Among tetracyclines, we revealed two drugs, doxycycline (82.4%, n = 28), and minocycline 17.6% (n = 6). Analyzing SRs with doxycycline, we found male gender in 96.4% (n = 27) and female in 3.6% (n = 1). Mean age was 45.3 ± 6.1 (min = 43, max = 80, median = 44, Q1 = 43, Q3 = 44, IQR = 1) years.

### 3.6. J01D Group

The group J01D includes antibacterials which are defined as “Other beta-lactams”; this group rounds out the top-five groups of antibacterials for systemic use identified in SRs with DIPF in our study (11.6%, n = 28). SR assessment revealed males in 89.3% (n = 25), females in 3.6% (n = 1), and no data on gender in 7.1% (n = 2). Mean age was 64.9 ± 6.2 (min = 31, max = 80, median = 65, Q1 = 65, Q3 = 65, IQR = 0) years. Most SRs resulted in unknown outcomes (67.9%, n = 19), death was reported in 28.6% (n = 8), and recovery without consequences was seen in 3.6% (n = 1). In 92.9% (n = 26) of SRs, cefuroxime was detected, and in 7.1% (n = 2), ceftriaxone. Demographic characteristics derived from SRs with cefuroxime are as follows: males were 92.3% (n = 24), and no information on gender was in 7.7% (n = 2); mean age was 65.4 ± 1.6 (min = 65, max = 72, median = 65, Q1 = 65, Q3 = 65, IQR = 0) years.

### 3.7. Disproportionality Analysis Results

A disproportionality analysis was conducted to identify drug–AE pairs exhibiting statistically elevated reporting frequencies for DIPF in association with the specified drug within the ATC J01 group. PRR and ROR values, along with their corresponding 95% CIs, were calculated for each of the top-five antibacterials detected in the total structure of J01 drugs.

**Figure 4:**
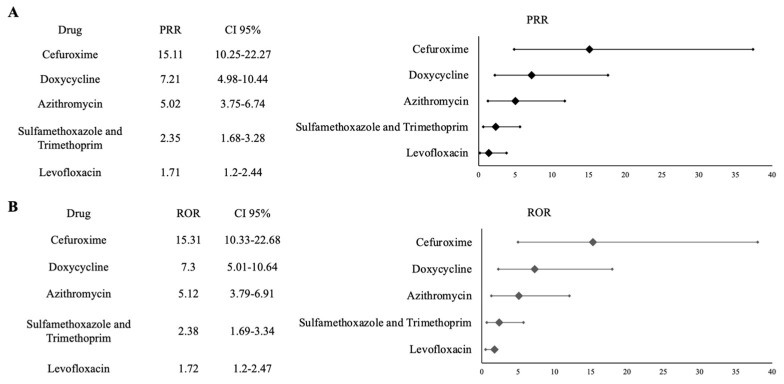
PRR and ROR analysis for the top-five antibacterials involved in DIPF. (**A**) PRR analysis data; (**B**) ROR analysis data.

PRR calculation revealed notable safety signals for DIPF across several antibacterial agents. Cefuroxime demonstrated the strongest association (PRR = 15.11, 95% CI: 10.25–22.27), followed by doxycycline (PRR = 7.21, 95% CI: 4.98–10.44) and azithromycin (PRR = 5.02, 95% CI: 3.75–6.74). Detailed data regarding PRR analysis performed for the top-five antibacterials are presented in Figure 4A and Table S2.

ROR analysis for the top-five J01 drugs associated with DIPF revealed definite signals only for cefuroxime (ROR = 15.31, 95% CI: 10.33–22.68), doxycycline (7.3, 95% CI: 5.01–10.64), and azithromycin (5.12, 95% CI: 3.79–6.91). Detailed results of ROR analysis for the top-five antibacterials are also given in Figure 4B and Table S3.

A primary limitation of the employed methods is the small sample size, necessitating cautious interpretation of findings and underscoring the need for additional studies with larger cohorts.

## 4. Discussion

DIPF constitutes an emerging and increasingly significant challenge in pharmacotherapy, as evidenced by the steadily rising number of AE reports recorded in major pharmacovigilance databases. Analysis of the FAERS database from 2000 to 2022 identified 17,520 cases of PF among 24 million reported AEs (0.07%), with reporting rates demonstrating exponential growth (R^2^ = 0.88) [3]. Similarly, the Russian National Pharmacovigilance database documented 1308 spontaneous reports on DIPF between 2019 and 2024, with case numbers nearly doubling annually [4]. It was shown that the incidence of drug-induced ILD ranges from 4.1 to 12.4 cases per million per year at the population level, with 3.0–6.4% PF cases. Mortality rates in PF are substantial, exceeding 50% in severe presentations, which supports our results [3,4].

Antibacterial agents may substantially contribute to DIPF development [5]. Among antibacterial agents, nitrofurantoin has the most well-documented profibrotic effect [11,28]. Mechanisms underlying nitrofurantoin-induced PF development include nitroreductase activation, which consequently generates reactive oxygen species (ROS) and induces lipid peroxidation, proto-myofibroblastic activation, enhanced fibronectin deposition, and oxidative damage via lymphocyte-mediated cytokine release [11]. Beta-lactam antibiotics have been demonstrated to exert profibrotic effects primarily through the induction of immune-mediated hypersensitivity reactions [29]. However, it must be noted that our findings partially contradict the documented anti-inflammatory and antifibrotic properties of these agents, thereby underscoring the need for more comprehensive investigations leveraging real-world data.

Based on frequency of occurrence in SRs, the five most commonly reported antibacterial agents were sulfamethoxazole (used alone or in combination with trimethoprim, 20.7%), azithromycin (18.2%), levofloxacin (12.4%), doxycycline (11.6%), and cefuroxime (10.7%). However, when applying disproportionality analysis with pre-specified signal detection criteria, only three of these agents demonstrated statistically disproportionate reporting: cefuroxime, doxycycline, and azithromycin.

### 4.1. Cefuroxime

Among the toxic reactions associated with cefuroxime, ophthalmic toxicity is the most well-documented, including retinal toxicity and maculopathy [30,31]. The nature of this reaction is explained by a reduction in esterase activity and membrane integrity disruption of retinal vascular cells [32]. According to Miyake H. et al. (2019), cefuroxime caused a significant increase of IL-8; IL-1ß, in in vitro studies, was seen to activate DAMP-induced inflammasomes [32]. IL-8 is a potent proinflammatory cytokine that induces inflammation via the CXCR1/CXCR2 pathway and activates neutrophils, monocytes, and lymphocytes, as well as increases TNF-α synthesis. This may potentially explain other toxic effects, such as hepatotoxicity and kidney damage, and provide insight into its potential for drug-induced pulmonary damage [33,34,35,36]. Published data indicated cefuroxime as a cause of toxic epidermal necrolysis, suggesting its ability to induce oxidative stress and to activate TNF-α [37]. We have found no direct causal relationship between cefuroxime and PF development in the existing literature; however, existing toxicity profiles related to other organs suggest a certain degree of systemic effect, which may also affect lung tissue.

### 4.2. Doxycycline

Doxycycline is known to induce hepatic and pancreatic injury [38]. According to El-Sanhoury et al. (2021), doxycycline may cause cytoplasmic vacuolization, multifocal hepatic necrosis with mononuclear infiltration and connective tissue proliferation in a rat model [39]. Doxycycline-induced cardiac toxicity results in loss of cardiomyocyte striation with subsequent myocardial necrosis accompanied by infiltration with mononuclear cells [39]. The precise mechanism of doxycycline-induced toxic reactions is unclear; however, existing evidence indicates that it generates ROS that may mediate potential multiorgan damage [40,41]. According to Nanda N. et al. (2016), doxycycline may contribute to chronic inflammation [42]. Doxycycline has been demonstrated to exacerbate granulomatous inflammation and induce remodeling of pulmonary tissue in patients infected with Schistosoma mansoni [43].

Although doxycycline has been demonstrated to exhibit profibrotic properties, there are studies reporting its antifibrotic effects, which are mainly mediated through non-selective inhibition of matrix metalloproteinases (MMP-2 and MMP-9), leading to reduced collagen deposition and attenuation of the fibrotic microenvironment [44]. In a bleomycin-induced model of PF, doxycycline significantly suppressed TGF-β1, connective tissue growth factor (CTGF), and type I collagen expression, while also preventing FN accumulation in renal tubular cells in ischemia–reperfusion injury models [45,46]. At sub-antimicrobial doses (20 mg twice daily), doxycycline reduced high-sensitivity CRP by 46% and pro-MMP-9 activity by 50% compared to placebo in the MIDAS pilot trial [47]. It must be emphasized, however, that the preponderance of evidence supporting the antifibrotic effects of doxycycline derives from studies employing murine models [45,48,49]. Such findings, while promising, cannot be reliably extrapolated to human physiology due to fundamental interspecies differences in disease pathogenesis, pharmacokinetics, and fibrotic responses [45,48,49].

Finally, we can state that the dual effects of doxycycline may be concentration- and context-dependent. At sub-antimicrobial doses (e.g., 20 mg twice daily in the MIDAS trial), doxycycline exerts antifibrotic effects primarily through non-selective inhibition of matrix metalloproteinases (MMP-2, MMP-9) and suppression of TGF-β1 signaling. However, at standard antimicrobial doses (100–200 mg daily), doxycycline generates reactive oxygen species (ROS) via mitochondrial dysfunction in susceptible cell types. Chronic ROS production can overwhelm local antioxidant defenses, leading to lipid peroxidation, DNA damage, and activation of latent TGF-β1—a potent pro-fibrotic driver. Additionally, doxycycline has been shown to exacerbate granulomatous inflammation and induce pulmonary tissue remodeling in the context of chronic infection (e.g., Schistosoma mansoni). Thus, the net effect of doxycycline on PF development may depend on dose, duration, underlying disease state, and individual susceptibility factors (e.g., genetic polymorphisms in antioxidant enzymes).

### 4.3. Azithromycin

Azithromycin is known to have a wide variety of AEs ranging from hearing loss to QT prolongation [50,51,52]. This happens due to its ability to accumulate within intracellular compartments in various cell types: hepatocytes, phagocytes, and white blood cells [53]. Jiang X. et al. (2019) revealed that azithromycin may upregulate gene expression of hypoxia-inducible factor 1 alpha (HIF1a) and cause DNA oxidative damage [53]. Its cardiac toxicity is of particular interest as it may provide insight into its potential lung damage. According to Salimi et al. (2016), azithromycin induces ROS formation, mitochondrial membrane permeabilization, and subsequent cytochrome release in a rat model [54]. It was also reported that azithromycin causes mitochondrial swelling in hepatocytes and Kupffer cells in another rat model [55]. The mechanism of drug-induced liver injury (DILI) is supposedly related to the charge of the N- atoms in the C5 position in the side chain group of the drug molecule, according to Zhang et al. (2022) [56].

Despite its potential for AE development, azithromycin possesses the most extensively characterized antifibrotic profile among the drugs reviewed, acting through proteasomal degradation of nicotinamide adenine dinucleotide phosphate oxidase 4 (NOX4) via the E3 ubiquitin ligase STUB1, thereby blocking TGF-β1-induced myofibroblast differentiation [57]. In a bleomycin mouse model, azithromycin significantly reduced expression of lysyl oxidase (LOX), lysyl oxidase-like 2 (LOXL-2), α-smooth muscle actin, and type I collagen through suppression of the TGF-β1/SMAD and JNK/c-Jun signaling pathways [58]. Azithromycin has also been identified as a senolytic agent that preferentially eliminates senescent fibroblasts (~97% clearance), thereby removing a major source of pro-fibrotic senescence-associated secretory phenotype (SASP) mediators [57,58].

Summarizing available data, we may conclude that azithromycin may produce various effects on fibroblasts, resulting in a decrease in their number. However, azithromycin also accumulates in macrophages and epithelial cells, where it can induce mitochondrial membrane permeabilization, cytochrome release, and ROS generation in these cell types. In the early or acute phase of lung injury—prior to established fibrosis—such ROS production could exacerbate epithelial injury, trigger inflammasome activation (IL-1β, IL-18), and promote a pro-fibrotic microenvironment. Azithromycin-induced mitochondrial dysfunction in type II pneumocytes may be supposed to be a factor impairing their regenerative capacity, indirectly facilitating fibrotic remodeling. Thus, the same drug may be protective in the context of established, senescence-driven fibrosis but deleterious during the acute injury phase or in individuals with underlying mitochondrial vulnerabilities.

### 4.4. Sulfamethoxazole and Trimethoprim

Our study identified sulfamethoxazole and trimethoprim (TMP-SMX) as the most frequently reported antibacterial agent; however, disproportionality analysis revealed only a weak signal for its association with DIPF. Published data provide no clear evidence of the profibrotic action of TMP-SMX. According to a case report of Donnan M. et al. (2024) [59], TMP-SMX treatment resulted in acute respiratory distress syndrome (ARDS) that required consequent lung transplantation. The histopathologic findings on day sixty-two of admission showed preserved alveolar architecture and interstitial thickening by reactive fibroblast incorporation, which implies a potential inflammatory cause of lung damage [59]. In patients with already diagnosed IPF, TMP-SMX did not improve the condition [60]. According to Güzel Bayülken D. et al. (2018), TMP-SMX and its conjugates demonstrated a cytotoxic and genotoxic effect when applied to cultured fibroblast cells; however, extrapolation of this effect on DIPF remains speculative [61]. A clinical case presented by Chen C. et al. (2022) suggests a potential link between TMP-SMX consumption and systemic inflammatory response syndrome (SIRS), which implies its potential role in inflammation development [62]. Another clinical case suggests cytotoxic hypersensitivity type IV reaction development after TMP-SMX consumption, which may be a possible link to profibrotic action [63].

### 4.5. Levofloxacin

Levofloxacin was detected among antibacterials involved in DIPF according to our data, but PRR and ROR values indicated the weakest association of this drug with DIPF. Numerous articles indicate its antifibrotic and anti-inflammatory properties that are based either on a decrease in proinflammatory IL-6, IL-8, TNF-α, or an increase in IL-10 [64]. Several articles have mentioned cytotoxic and proapoptotic effects of levofloxacin on connective tissue of annulus fibrosus in a rat model and fibroblast-like synoviocytes in a rabbit model; however, this may still be related to antifibrotic action [65,66]. Some studies present potential mechanisms of levofloxacin toxicity based on its ability to generate ROS and upregulate the Bax/Bcl-2 gene ratio [67]. Chronic ROS production may consequently lead to oxidative stress, inflammation, and damage of pulmonary tissue, which may result in PF development [68,69].

Although existing evidence regarding the profibrotic or antifibrotic effects of antibacterials remains contentious, our analysis of SRs derived from the national pharmacovigilance database indicates that, where clinically suitable alternatives are available, clinicians should prioritize antibiotics associated with a lower suspected risk of DIPF, especially in patients with preexisting lung disease or other predisposing factors. In situations where high-risk agents such as cefuroxime, doxycycline, or azithromycin are necessary, their use should be accompanied by careful risk–benefit assessment, closer clinical monitoring for new respiratory symptoms, and a lower threshold for treatment discontinuation if pulmonary toxicity is suspected. Our findings underscore the need for targeted investigations using human cell lines to elucidate the potential mechanisms underlying the profibrotic effects of antibacterials. Larger-scale, multicenter clinical trials are warranted to estimate the precise role of antibacterials in the pathogenesis, progression, and management of DIPF. Such investigations should employ rigorous methodologies, including randomized controlled designs, standardized diagnostic criteria for DIPF, and comprehensive long-term follow-up to assess both efficacy and safety outcomes. By generating high-quality evidence, these studies hold the potential to inform evidence-based revisions to drug labeling, prescribing guidelines, and clinical protocols, thereby optimizing therapeutic strategies and minimizing iatrogenic risks for at-risk patient populations.

The apparent contradiction between pro- and antifibrotic reports for the same drug can be reconciled by considering: (1) dose dependency (sub-antimicrobial vs. standard doses), (2) timing of administration relative to injury phase (prevention vs. early injury vs. established fibrosis), (3) cell-type specificity (fibroblasts vs. epithelial cells vs. macrophages), (4) underlying disease context (normal lung vs. pre-existing subclinical ILD vs. acute infection), and (5) host factors (antioxidant capacity and genetic polymorphisms in drug-metabolizing enzymes). SRs cannot capture these nuanced determinants; thus, observational associations should not be equated with a fixed “pro-fibrotic” or “anti-fibrotic” classification for any given drug.

We should also emphasize that the apparent paradox of drugs with known antifibrotic properties appearing in DIPF reports may be explained by protopathic bias (antibiotics prescribed for early symptoms of unrecognized PF) or by context-dependent, concentration-dependent, or cell-type-specific biological effects. SR data cannot distinguish these possibilities. Therefore, our findings should not be interpreted as definitive evidence of pro-fibrotic drug effects, nor as refutation of potential anti-fibrotic properties. Rather, they highlight the need for prospective studies that incorporate detailed clinical phenotyping, longitudinal follow-up, and mechanistic investigations using human cell lines to clarify the conditions under which these drugs may either protect against or contribute to PF.

### 4.6. Limitations

The limitations of our study are common to those inherent in the spontaneous reporting method and include the following: retrospective nature of the study, risks of underreporting, fragmentary evidence, inconsistent reporting rates across various settings and time frames, the poor quality of some SRs, and the inability to determine the true frequency of ADRs due to the unknown total population of drug users.

Although all included SRs underwent Naranjo algorithm assessment, critical data elements were frequently missing: time to onset was recorded in only 17.7% of SRs, dechallenge information in 13.8%, and rechallenge in none. Alternative etiologies of PF—such as other medications (including antineoplastic or antirheumatic agents), pre-existing subclinical ILD, connective tissue diseases, or environmental/occupational exposures—were not systematically excluded. A further critical limitation of our study is the absence of data on the indications for which the suspected antibacterial agents were prescribed. So, we cannot exclude the possibility that in a subset of cases, PF may have been attributable to the underlying respiratory infection (e.g., pneumonia, bronchitis) rather than to the antibacterial agent itself. Our findings should therefore be interpreted as generating hypotheses—specifically, safety signals—that require rigorous validation in study designs that can control for the underlying disease, such as large-scale cohort studies or nested case–control studies with detailed clinical data.

## 5. Conclusions

The present study highlighted the contribution of antibacterial agents to the development of DIPF, revealing a consistent increase in SRs of antibacterial-associated PF between 2020 and 2025. Retrieved information revealed a strong signal detection for cefuroxime, while the top-five antibacterials with the highest frequency of occurrence among SRs with DIPF were sulfamethoxazole (used alone or in combination with trimethoprim), azithromycin, and levofloxacin. As the direct mechanisms underlying antibiotic-induced PF remain poorly documented in the literature, there is a pressing need for additional in vitro studies using human cell lines and clinical trials aimed on investigation of details of the pathogenesis of antibacterial-induced PF. Our study did not establish definitive causality; nonetheless, the safety signals detected for cefuroxime, doxycycline, and azithromycin highlight the need for validation through prospective and randomized study designs. The observed associations between antibacterials and DIPF may reflect protopathic bias or context-dependent effects rather than true causality, highlighting the need for prospective studies and mechanistic investigations to validate these safety signals.

## Figures and Tables

**Table 1:** Two-by-two contingency table.

	Reaction of Interest	All Other Reactions
Suspected Drug	a	b
All Other Drugs	c	d

a = Number of reports for the selected ATC J01 drug with DIPF. b = Number of reports for the selected ATC J01 drug with any other ADRs. c = Number of reports for all other drugs (including other J01) with PF. d = Number of reports for all other drugs with any other ADRs.

**Table 2:** Outcome analysis.

Outcome	N (Total = 130)	%
Death	65	50.0
Condition unchanged/Unknown	63	48.4
Recovery without consequences	1	0.8
Condition improved	1	0.8

**Table 3:** Number of antibacterials of the group J01 per SR.

N of J01 Drugs	N of SRs (Total = 130)	%
1	66	50.8
2	20	15.4
3	21	16.2
4	14	10.8
5	4	3.1
6	3	2.3
7	1	0.8
9	1	0.8

**Table 4:** Structure of J01 subgroups.

Drug Group	ATC	N (Total = 242)	%
Sulfonamides and trimethoprim	J01E	65	26.9
Quinolone antibacterials	J01M	50	20.7
Macrolides, lincosamides, streptogramines	J01F	48	19.8
Tetracyclines	J01A	34	14.0
Other beta-lactams	J01D	28	11.6
Beta-lactams, penicillins	J01C	13	5.4
Other antibacterials	J01X	3	1.2
Amphenicols	J01B	1	0.4

**Table 5:** The structure of drugs within the J01E group.

J01E Group	ATC	N (Total = 65)	%
Sulfamethoxazole and Trimethoprim	J01EE01	34	52.3
Sulfamethoxazole	J01EC01	16	24.6
Sulfadiazine	J01EC02	8	12.3
Sulfathiazole	J01EB07	5	7.7
Sulfisomidine	J01EB01	1	1.5
Sulfadiazine and Trimethoprim	J01EE02	1	1.5

## Data Availability

The original contributions presented in this study are included in the article/Supplementary Material. Further inquiries can be directed to the corresponding author.

## Supplementary Materials

The following supporting information can be downloaded at: https://www.mdpi.com/article/10.3390/biomedicines14061182/s1, Table S1. Overall J01 drug structure. Table S2. PRR analysis for the top-five antibacterials involved in DIPF. Table S3. ROR analysis for the top-five antibacterials involved in DIPF.

## Abbreviations

The following abbreviations are used in this manuscript:

AE	Adverse Event
ADR	Adverse Drug Reaction
AIS	Automated Information System
AR	Adverse Reaction
ARDS	Acute Respiratory Distress Syndrome
ATC	Anatomical Therapeutic Chemical Classification System
CI	Confidence Interval
CTGF	Connective Tissue Growth Factor
DILI	Drug-Induced Liver Injury
DIPF	Drug-Induced Pulmonary Fibrosis
ECM	Extracellular Matrix
EAEU	Eurasian Economic Union
FAERS	Food and Drug Administration Adverse Event Reporting System
HIF1a	Hypoxia-Inducible Factor 1 Alpha
HLT	High-Level Term
ICH	International Council for Harmonisation of Technical Requirements for Pharmaceuticals for Human Use
ICH E2A	ICH Guideline E2A (Clinical Safety Data Management)
ICH E2B (R3)	ICH Guideline E2B (R3) Electronic Transmission of Individual Case Safety Reports
IL-1β	Interleukin-1 Beta
IL-6	Interleukin-6
IL-8	Interleukin-8
IL-10	Interleukin-10
ILD	Interstitial Lung Disease
INN	International Nonproprietary Name
IPF	Idiopathic Pulmonary Fibrosis
IQR	Interquartile Range
JNK	c-Jun N-terminal Kinase
LOX	Lysyl Oxidase
LOXL-2	Lysyl Oxidase-Like 2
MMP	Matrix Metalloproteinase
MMP-2	Matrix Metalloproteinase-2
MMP-9	Matrix Metalloproteinase-9
MIDAS	MIDAS pilot trial (full trial name not specified in manuscript)
NOX4	NADPH Oxidase 4
PF	Pulmonary Fibrosis
PRR	Proportional Reporting Ratio
PT	Preferred Term
ROR	Reporting Odds Ratio
ROS	Reactive Oxygen Species
SASP	Senescence-Associated Secretory Phenotype
SIRS	Systemic Inflammatory Response Syndrome
SR	Spontaneous Report
STUB1	STIP1 Homology and U-Box Containing Protein 1
TGF-β1	Transforming Growth Factor Beta 1
TNF-α	Tumor Necrosis Factor Alpha
TMP-SMX	Trimethoprim–Sulfamethoxazole
WHO	World Health Organization
