# Supplementary material for: Antibiotic-Induced Pulmonary Fibrosis: National Database Analysis
Source: Biomedicines. 2026 May 22;14(6):1182. doi: 10.3390/biomedicines14061182 (PMC13296167; doi:10.3390/biomedicines14061182)
Supplement: Supplementary file 1 [file biomedicines-14-01182-s001.zip › biomedicines-4322347-supplementary.pdf]

Supplementary materials

Table S1. Overall J01 drug structure.

J ANTIINFECTIVES FOR SYSTEMIC USE	ATC	N (total=242)	%
J01 ANTIBACTERIALS FOR SYSTEMIC USE			
J01A TETRACYCLINES			
J01AA Tetracyclines			
Doxycycline	J01AA02	28	11,6
Minocycline	J01AA08	6	2,5
J01B AMPHENICOLS			
J01BA Amphenicols			
chloramphenicol	J01BA01	1	0,4
J01C BETA-LACTAM ANTIBACTERIALS, PENICILLINS			
J01CA Penicillins with extended spectrum			
Amoxicillin	J01CA04	5	2,1
J01CR Combinations of penicillins, incl. beta-lactamase inhibitors			
Amoxicillin+Clavulanic acid	J01CR02	8	3,3
J01D OTHER BETA-LACTAM ANTIBACTERIALS			
J01DC Second-generation cephalosporins			
Cefuroxime	J01DC02	26	10,7
J01DD Third-generation cephalosporins			
Ceftriaxone	J01DD04	2	0,8
J01E SULFONAMIDES AND TRIMETHOPRIM			
J01EB Short-acting sulfonamides			
Sulfisomidine	J01EB01	1	0,4
Sulfathiazole	J01EB07	5	2,1
J01EC Intermediate-acting sulfonamides			
Sulfamethoxazole	J01EC01	16	6,6
Sulfadiazine	J01EC02	8	3,3
J01EE Combinations of sulfonamides and trimethoprim, incl. derivatives			
Sulfamethoxazole and Trimethoprim	J01EE01	34	14,0
Sulfadiazine and Trimethoprim	J01EE02	1	0,4
J01F MACROLIDES, LINCOSAMIDES AND STREPTOGRAMINS			
J01FA Macrolides			
Erythromycin	J01FA01	4	1,7
Azithromycin	J01FA10	44	18,2
J01M QUINOLONE ANTIBACTERIALS			
J01MA Fluoroquinolones			
Levofloxacin	J01MA12	30	12,4
Moxifloxacin	J01MA14	20	8,3
J01X OTHER ANTIBACTERIALS			
J01XD Imidazole derivatives			
Metronidazole	J01XD01	3	1,2

Table S2. PRR analysis for the top-5 antibacterials involved in DIPF.

Drug	PRR	CI 95%
Cefuroxime	15.11	10.25-22.27
Doxycycline	7.21	4.98-10.44
Azithromycin	5.02	3.75-6.74
Sulfamethoxazole and Trimethoprim	2.35	1.68-3.28
Levofloxacin	1.71	1.2-2.44

Table S3. ROR analysis for the top-5 antibacterials involved in DIPF.

Drug	ROR	CI 95%
Cefuroxime	15.31	10.33-22.68
Doxycycline	7.3	5.01-10.64
Azithromycin	5.12	3.79-6.91
Sulfamethoxazole and Trimethoprim	2.38	1.69-3.34
Levofloxacin	1.72	1.2-2.47
